# Supplementary material for: Defective PITRM1 mitochondrial peptidase is associated with Aβ amyloidotic neurodegeneration
Source: EMBO Mol Med. 2015 Dec 23;8(3):176–90. doi: 10.15252/emmm.201505894 (PMC4772954; doi:10.15252/emmm.201505894)
Supplement: Supplementary file 1 — Appendix [file EMMM-8-176-s001.pdf]

Appendix Material Online

CONTENTS

1. **Appendix Supplementary Methods**
2. **Appendix Figures S1-S5 and corresponding legends**
3. **Appendix Tables S1 & S2**
4. **Appendix References**

Appendix Supplementary Methods

Purification of DNA and RNA.

Genomic DNA was purified from EDTA-blood using the QiaSymphony instrument (Qiagen, Hilden, Germany). Mitochondrial DNA was extracted from $5x10^5$ cells using the Qiagen, QIAamp DNA Mini Kit (50, #51304) as described by the manufacturer. Mitochondrial DNA was quantified and deletions analysed using qPCR as described previously (Tzoulis et al., 2013). For RNA, $1x10^5$ cells per well were seeded in 6-well plates and cultured for 48 hours. Cells were washed twice with PBS, lysed and total mRNA isolated using the RNeasy MiniKit (Qiagen, Hilden, Germany).

Cell culture

Fibroblasts from a skin biopsy taken for diagnostic purposes were grown under standard conditions either in supplemented high glucose DMEM (Sigma) or in glucose-free DMEM with the addition of 10% dialysed foetal bovine serum and 5 mM galactose. Primary fibroblasts were immortalized by lentiviral transduction using the pLox-Ttag-ires-TK vector (Tronolab). Growth curves were assessed using an Incucyte ZOOM instrument (Essen Bioscience) using an algorithm to calculate cell confluency based on microscope imaging of the plates. Images were taken every six

hours for a total period of 5 days. Doubling time calculations and statistical analysis were performed using the GraphPad PRISM version 5.0 software.

RNA studies

50 ng mRNA (2.5 ng/ul) was reverse-transcribed using the High-Capacity cDNA Reverse Transcriptase Kit (ABI Biosystems, Foster City, CA). Real-time quantitative PCR was performed using the TaqMan Fast Advanced master mix with *PITRM1* primers and probes from the Taqman gene expression assays (Applied Biosystems). The housekeeping gene *PLP0* was used as loading control and the delta-delta CT method was used to calculate the expression of the genes of interest in the patient relative to healthy controls.

For the RNAi assays, five different MISSION® shRNA lentiviral constructs targeting human *PITRM1* mRNA: TRCN0000052238-42, were purchased from Sigma-Aldrich. All five were tested in control, immortalized fibroblasts, being TRCN0000052238, TRCN0000052241 and TRCN0000052240 the most efficient, in this order, in knocking-down PITRM1 expression..

Biochemical analysis of the mitochondrial Respiratory Chain

The spectrophotometric activity of complex I, complex II, complex III, and complex IV, as well as citrate synthase, were measured as described (Bugiani et al., 2004). Note that in all panels the activity of complex II has been multiplied by 10 for visualization clarity.

Antibodies and Western Blot analysis

Thirty micrograms of proteins were used for each sample in denaturing sodium dodecyl sulphate – polyacrylamide gel electrophoresis (SDS – PAGE). Western blot analysis was performed using the ECL-chemiluminescence kit (Amersham). The following rabbit polyclonal antibodies were used: anti-PITRM1 (Atlas), anti-

VDAC1 (Abcam) at 1:1000 dilution; anti-LONP1 (Abcam); anti-HSP60 (Abcam); anti-Grp75 (Abcam); anti-CLPP (Atlas). Mouse monoclonal anti-GAPDH (Abcam), anti-Porin antibody (Abcam); anti-TOMM20 (Abcam) were used as loading controls. Anti-*myc* antibody was from Sigma.

To detect beta-amyloid accumulation, protein concentration was quantified from isolated mitochondria according to Bradford (Bio-rad). Mitochondrial proteins were then separated by SDS-PAGE, and electro-blotted onto nitrocellulose filters, which were then incubated with anti-Aβ1-42 (6E10, Biolegend) and anti-Porin (AbcamMitoscience) as a loading control. To quantify $Cym1^{wt}$ and $cym1^{R163Q}$ we used total protein extracts of strains expressing HA-tagged recombinant variants, and the filters were incubated with anti-HA (Roche Applied Science) and anti-PGK (Abcam Mitoscience).

Oligonucleotides, plasmids, cloning, mutagenesis and transformation

Oligonucleotides used in this work are reported in Supplementary Table 2.

$CYM1^{wt}$ was PCR amplified with its promoter and its terminator with primers CYM1DCFw and CYM1DCRv. The fragment was digested with *Kpn*I and *Pst*I, cloned in pFL38 and sequenced. The $cym1^{R163Q}$ mutant allele was constructed by mutagenic overlap PCR using primers CYM1CFw, CYM1R163QRv, CYM1R163QFw and CYM1MS2, and cloned in *KpnI-SacI*-digested pFL38CYM1 plasmid. Both wt and mutant alleles were subcloned in pFL39. pYES2-mt$A\beta_{1\text{-}42}^{myc}$ (a kind gift from Roberta Ruotolo and Simone Ottonello) contained a DNA fragment encoding the mitochondrial peptide signal of Sod2 in frame with the myc peptide and with the $A\beta_{1\text{-}42}$ peptide. The HA-tagged $Cym1^{wt}$ and $cym1^{R163Q}$ recombinant proteins were obtained adding the HA epitope at the C-terminal by PCR overlap using primers CYM1M2Fw, CYM1HARv, CYM1HAFw and CYM1CPstIRv and cloning the fragment in S*alI-PstI*-digested pFL38CYM1 plasmid. Transformation of W303-1B *cym1Δ* with

plasmid-born tagged or untagged *CYM1*^{wt} or *cym1*^{R163Q} and with pYES2-mtAβ1-42^{myc} was performed after growth in YPAD medium (1% Yeast extract (Formedium), 2% Peptone (Formedium), 40 mg/l adenine base and 2% glucose) according to (Gietz and Woods, 2002).

Cytochrome spectra, mitochondrial respiration and respiratory chain complex activities.

Reduced vs. oxidized spectra were recorded using a Varian Cary300 UV-VIS Spectrophotometer at room temperature, from suspensions of yeast cells cultured for 18 hrs at 28˚C in SC medium supplemented with 0.5% glucose until exhaustion. Oxygen consumption rate (OCR) was measured at 30°C from the same suspensions using a Clark-type oxygen electrode (Oxygraph System Hansatech Instruments England) with 1 ml of air-saturated respiration buffer (0.1 M phthalate–KOH, pH 5.0), 0.5% glucose. The reaction started by addition of 20 mg of wet-weight cells. Cytochrome c oxidase (CIV), NADH-cytochrome c oxidoreductase activity NCCR (complex III) and Succinate Quinone DCPIP Reductase SQDR (complex II) activities were measured in a mitochondrial-enriched fraction prepared as previously described[37] after a cellular growth to a concentration of 1.5-2 OD_{600} in SC medium supplemented with 0.5% glucose until exhaustion.

Statistical analysis was performed by unpaired two-tailed Student's t-test.

Behavioural and locomotor analysis. Mice were monitored weekly for onset of postural abnormalities, weight loss and general health. No significant weight differences were found between the two groups. The first evidence of abnormality was the development of hindlimb feet clasping in heterozygous *Pitrm1*$^{+/-}$ male mice from the age of 2 months. Neurological phenotype was evaluated further with a set of different coordination and sensorimotor tests.

<u>Rotarod</u>*:* to measure coordination skills, a Rotarod (Ugo Basile, Italy) apparatus was

set with a starting velocity of 4 rpm and an acceleration of 20 rpm/min. All of the animals received 3 days of training consisting of three trials for each session. On the test day, animals received 3 trials and the time of latency to fall was calculated for each mouse.

Cylinder Test: to measure rearing activity, animals were placed in a clear Plexiglas cylinder (13.5 cm inside diameter x 17.6 cm height). While in the cylinder, animals typically rear and engage in exploratory behavior by placing their forelimbs along the wall of the cylinder. Activity was measured by counting the number of rears made by each animal in a 3-min period without recording specific limb use.

Pole test*:* Animals were placed head upwards on top of a vertical plastic pole 50 cm in length (diameter, 0.5 cm). The base of the pole was placed in the home cage. Once placed on the pole, normal animals orientate themselves downward and descend the length of the pole back into their home cage. All of the animals received 2 days of training consisting of three trials for each session. On the test day, animals received 3 trials and the time to orientate downward and descend the pole was scored as follows: 0 = Falls off; 1 = Turns, but falls off; 2 = Turns and climbs down within 25s; 3 = Turns and climbs down within 15s; 4 = Turns and climbs down within 10s

Negative geotaxis test*:* this test assesses the ability of the mouse to turn on a vertically positioned grid from being in the head-down position to the head-up position by rotating the body by 180°. Some mice may not be able to maintain their position on the grid at all (scored as 0), or may freeze for the duration of the 30 s test (1), move slightly but do not turn (2), turn and freeze (3) or turn and climb the grid (4).

Comprehensive laboratory animal monitoring system (CLAMS)*:* the Comprehensive Laboratory Animal Monitoring System (CLAMS™, Columbus Instruments, Columbus, OH) is a set of live-in cages for automated, non-invasive and simultaneous monitoring of horizontal and vertical activity, feeding and drinking, O_2 consumption and CO_2 production. Seven *Pitrm1*$^{+/-}$ male mice and four control male littermates

were individually placed in CLAMS™ cages and monitored over a 36 hr period. Food and water consumption are measured directly as accumulated data. The following parameters were measured: VO_2 (volume of oxygen consumed, mL/Kg/hr), VCO_2 (volume of carbon dioxide produced, mL/Kg/hr), RER (respiratory exchange ratio), heat (Kcal/hr), XY total activity (all horizontal beam breaks in counts); data were collected every 10-minutes.

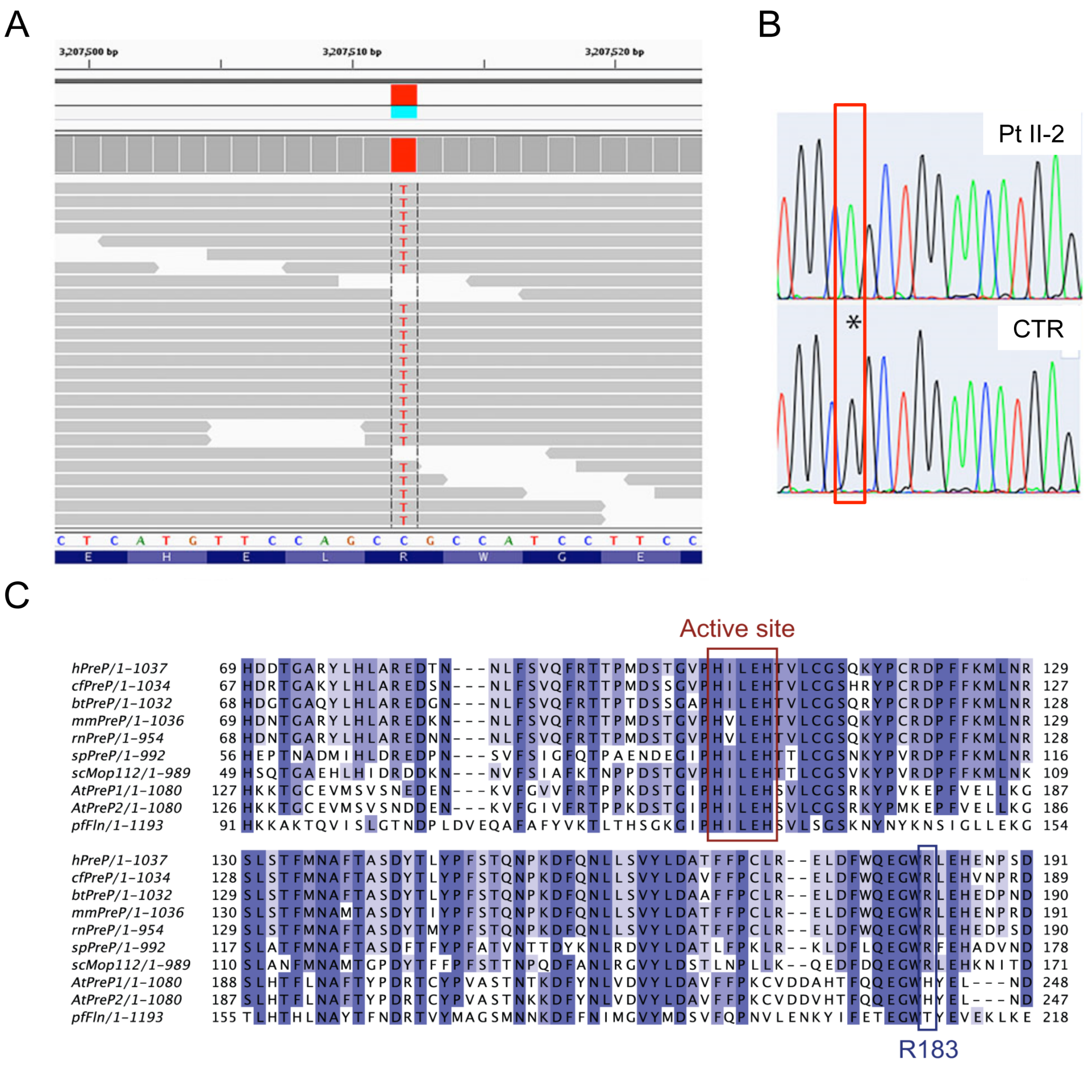

Appendix figure S1. Sequencing overview of the *PITRM1* c.548G>A, p.Arg183Gln mutation.

Whole exome sequencing to a mean 100X coverage was performed in the index patient II-2. There were 20,436 genetic variants of which 240 were coding and not found in our in-house frequency database or in the 1000 Genomes database at

>0.5% allele frequency. A total of 13 genes contained rare variants consistent with autosomal recessive inheritance and of these, only one gene, *PITRM1*, was located within a homozygous region shared by both affected siblings. The mutation NM_014889.2:c.548G>A, p.Arg183Gln was verified by Sanger sequencing.

A: IGV screenshot of mutation in proband.

B: Sanger sequencing confirmed the homozygous mutation in both affected individuals. Shown are the electropherograms of the affected proband (top) and one healthGy control (bottom). The asterisk and the red box highlight the changed nucleotide.

C: Protein alignment from several species showing the conservation of the R183 residue (blue box) and its relationship to the active site of the enzyme (red box).

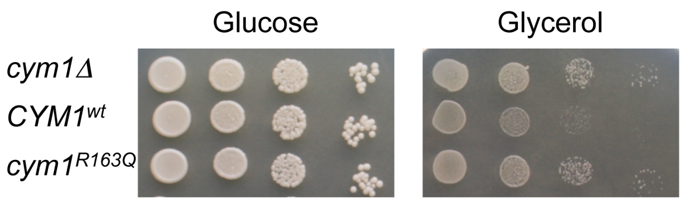

Appendix figure S2. Oxidative growth of yeast strains at 28°C.

Note that growth of *cym1Δ* and *cym1*^{R163Q} mutant strains is comparable to that of *CYM*^{wt} control strain in both glucose and glycerol media. Compare with Figure 3A (growth at 37°C).

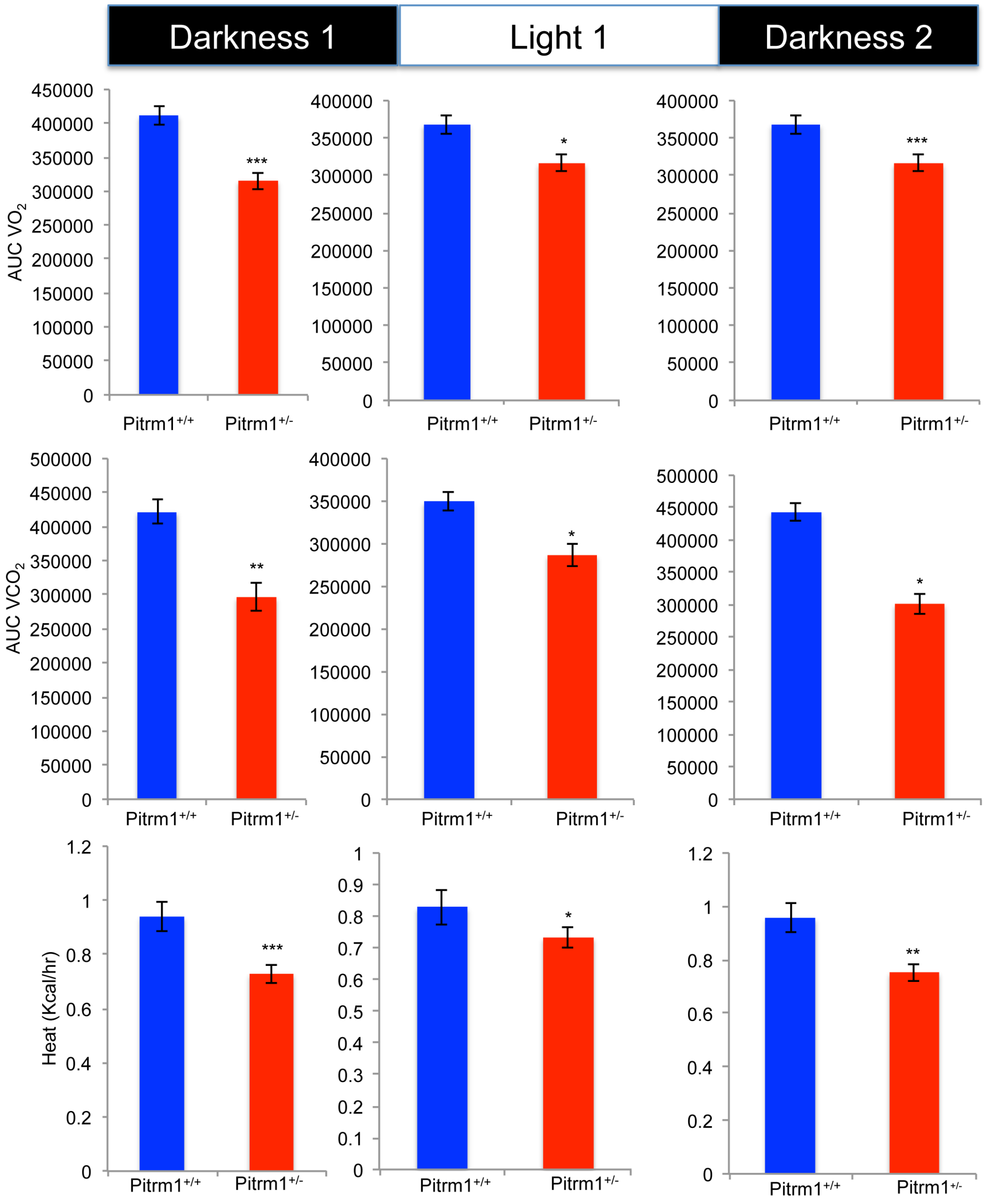

Appendix figure S3. Metabolic characterization of Pitrm1$^{+/-}$ mice.

Experiments were carried out in a comprehensive laboratory animal monitoring system (CLAMS).

Graphs report (in arbitrary units) the volume of oxygen (VO_2) and volume of CO2 (VCO_2) consumed as well as the heat production, measured during a 24 hr period with three alternate cycles of darkness vs. light. Blue bars refer to 6 mo Pitrm1$^{+/+}$ males (n. 9), red bars to Pitrm1$^{+/-}$ male littermates. Statistic analysis was by unpaired, two-tail Student's *t* test. * p<0.05; ** p<0.01; *** p<0.001.

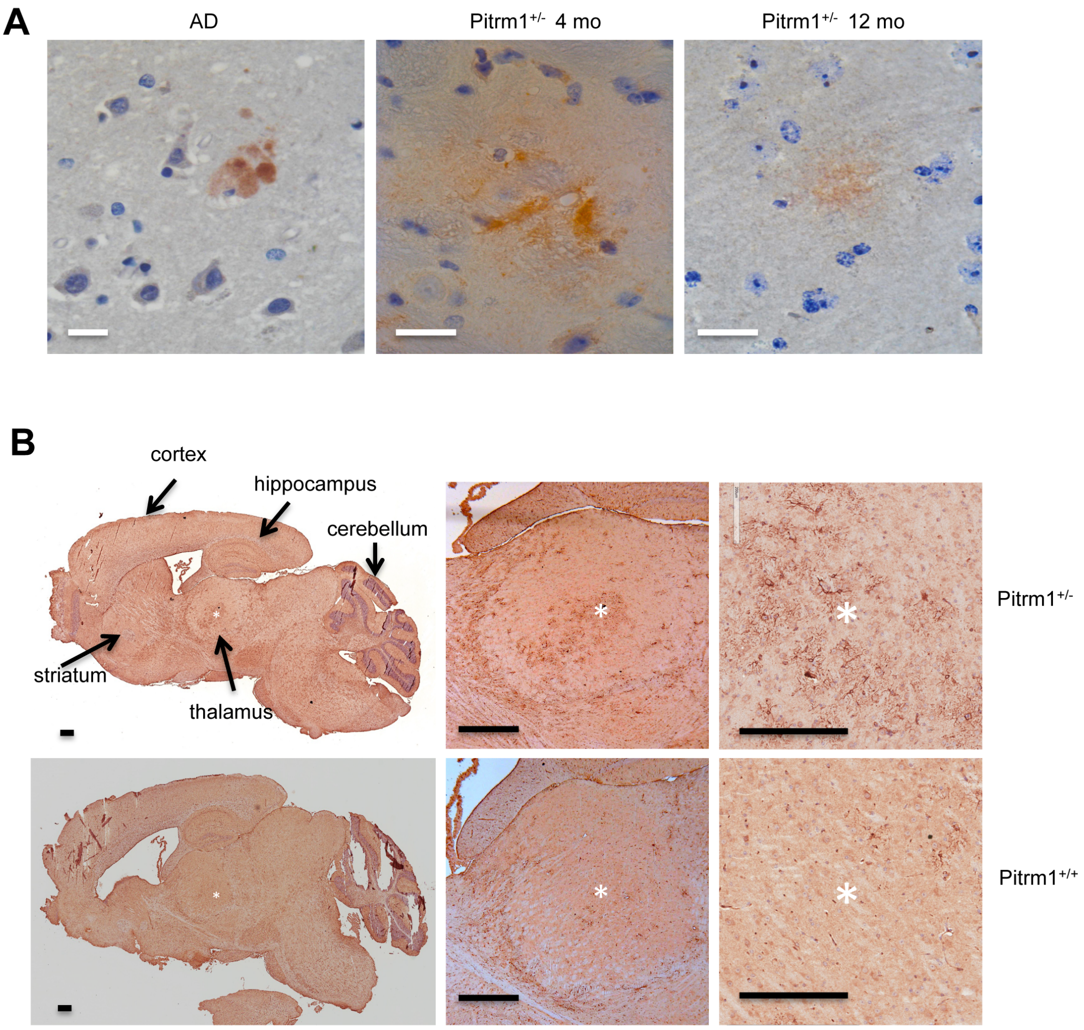

Appendix figure S4. Additional morphological studies in *Pitrm1*$^{+/-}$ mouse brains.

A: Amyloid-precursor protein (APP) immunostaining shows granular deposits in an AD subject and in *Pitrm1*$^{+/-}$ mouse brains of 4 and 12 months.

B: Glial fibrillary acidic protein (GFAP) immunostaining highlights areas of gliosis (white asterisks) only in the *Pitrm1*$^{+/-}$ mouse brains (age 12 months).

The black bar indicates 500 µm. A white asterisk indicates the thalamus.

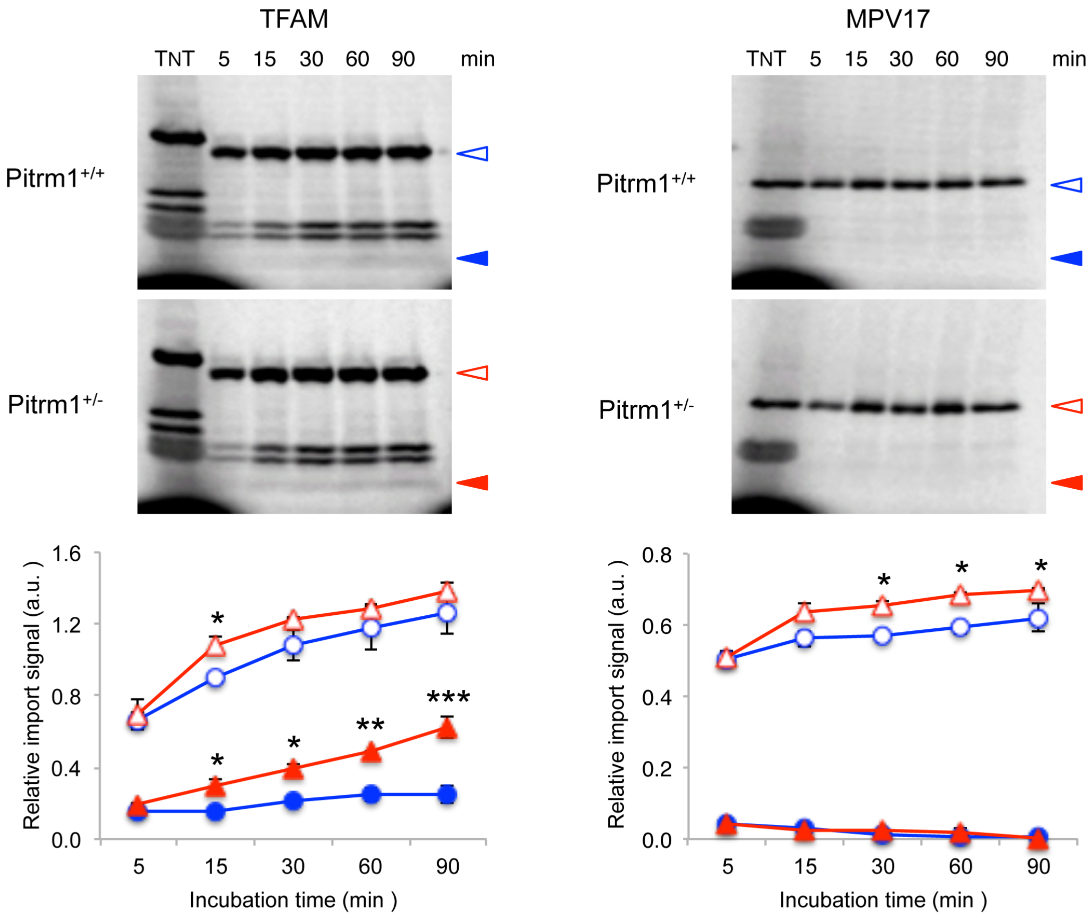

Appendix figure S5. Mitochondrial signal peptide degradation.

Import of TFAM (left) and MPV17 (right) into isolated liver mitochondria from *Pitrm1*$^{+/+}$ and *Pitrm1*$^{+/-}$ mice. Quantification from two different experiments of both the mature protein (open triangles) and signal peptide (close triangles) are related to the TNT signal. Note the accumulation of the low molecular band corresponding to the cleaved leader peptide in TFAM but not MPV17 samples. Two-tailed unpaired Student's *t* test*:* *p<0.05; **p<0.01; ***p<0.001. Error bars indicate ±SD.

Appendix Table S1. Yeast strains used in the study

Strain	Genotype	Source
W303-1B	*Matα ade2-1 leu2-3, 112 ura3-1 trp1-1 his3-11, 15 can1-100*	Thomas and Rothstein, 1989
W303-1B *Δcym1*	*Matα ade2-1 leu2-3, 112 ura3-1 trp1-1 his3-11, 15 can1-100 cym1*::KanMX4	This study
BY4742 *Δcym1*	*Matα his3Δ1 leu2Δ0 lys2Δ0 ura3Δ0 cym1::KanMX4*	Yeast Deletion Project

Appendix Table S2. Oligos used in the yeast studies

Name	Sequence
CYM1DCFw	GGGGCGGTAccttatggttttagcgtaattgc
CYM1DCRv	CGGGGCTGCAggtctttggtcttttgatcc
CYM1S1Fw	tgagcagttcagtggttacg
CYM1MS2Rv	cacaaatccacgtcattgagg
CYM1S3Fw	gaagatttatcctgtttacctacc
CYM1R163QFw	gatcaggagggttggCAgCtggagcataaaaacatcacagac
CYM1R163QRv	gtctgtgatgtttttatgctccaGcTGccaaccctcctgatc
CYM1HARv	atctaAGCGTAATCTGGAACATCATATGGATATCCACCcagttccttcacctcccaatttg
CYM1HAFw	actgGGTGGATATCCATATGATGTTCCAGATTACGCTtagatatataatttaatcctg
CYM1M2Fw	cttcaaacacatcttctgtagc

Appendix References

Bugiani, M., Invernizzi, F., Alberio, S., Briem, E., Lamantea, E., Carrara, F., Moroni, I., Farina, L., Spada, M., Donati, M.A., et al. (2004). Clinical and molecular findings in children with complex I deficiency. Biochimica et biophysica acta *1659*, 136-147.

Gietz, R.D., and Woods, R.A. (2002). Transformation of yeast by lithium acetate/single-stranded carrier DNA/polyethylene glycol method. Methods in enzymology *350*, 87-96.

Tzoulis, C., Tran, G.T., Schwarzlmuller, T., Specht, K., Haugarvoll, K., Balafkan, N., Lilleng, P.K., Miletic, H., Biermann, M., and Bindoff, L.A. (2013). Severe nigrostriatal degeneration without clinical parkinsonism in patients with polymerase gamma mutations. Brain : a journal of neurology *136*, 2393-2404.
