# Supplementary material for: Defective PITRM1 mitochondrial peptidase is associated with Aβ amyloidotic neurodegeneration
Source: EMBO Mol Med. 2015 Dec 23;8(3):176–90. doi: 10.15252/emmm.201505894 (PMC4772954; doi:10.15252/emmm.201505894)
Supplement: Supplementary file 2 — Expanded View Figures PDF [file EMMM-8-176-s002.pdf]

Expanded View Figures

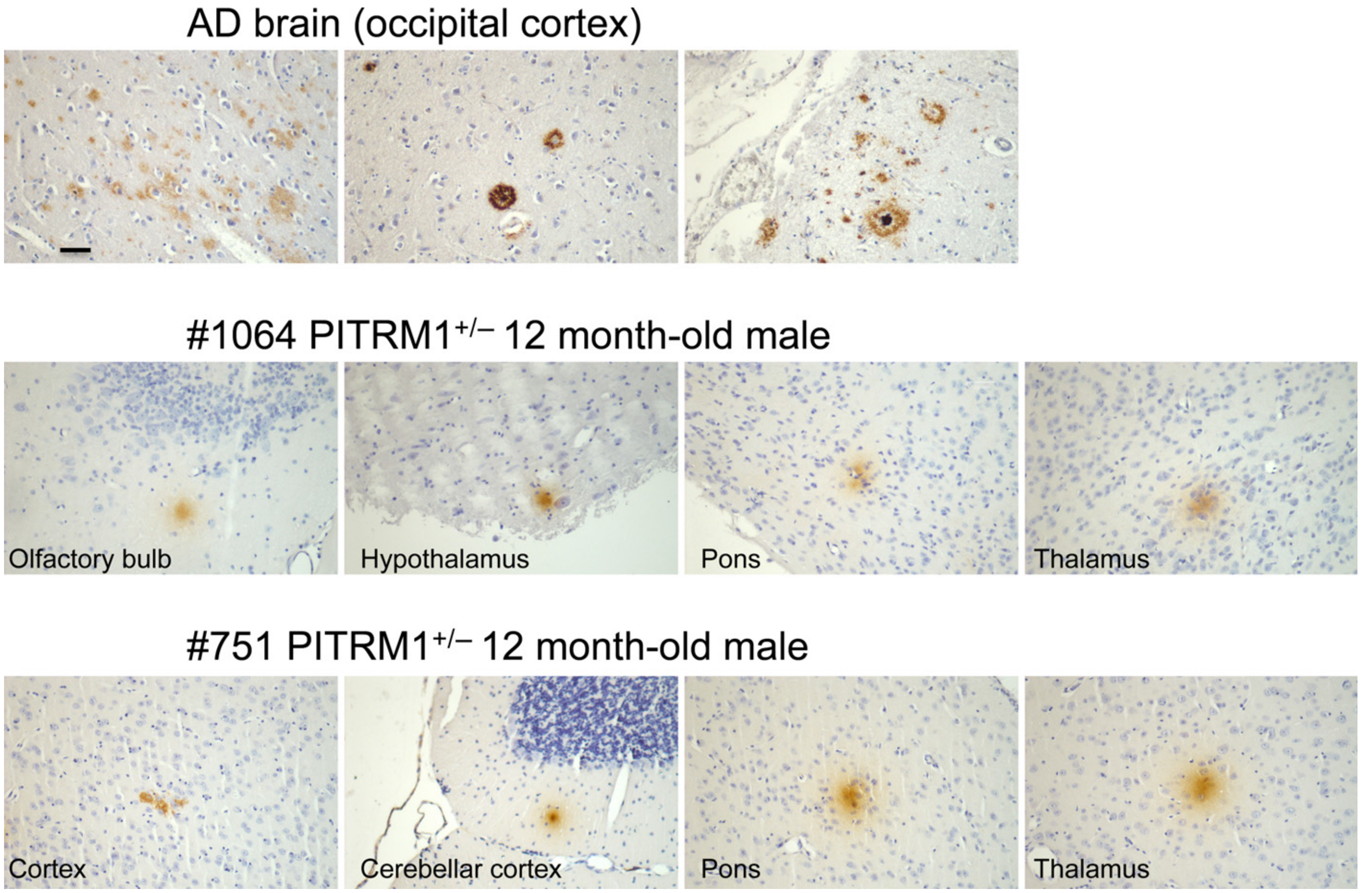

Figure EV1. Aβ_{1-42} immunostaining in brains of 12-mo mice.
Brain sections from an AD patient are shown as positive controls. The brownish areas correspond to Aβ_{1-42} deposits. A black bar indicates 100×. The same magnification was used for all images.

 © 2015 The Authors
