# Supplementary material for: Defective PITRM1 mitochondrial peptidase is associated with Aβ amyloidotic neurodegeneration
Source: EMBO Mol Med. 2015 Dec 23;8(3):176–90. doi: 10.15252/emmm.201505894 (PMC4772954; doi:10.15252/emmm.201505894)
Supplement: Supplementary file 5 — Source Data for Figure 1E and G [file EMMM-8-176-s004.pdf]

RAW DATA FIG. 1 E FIBROBLAST

WB #1

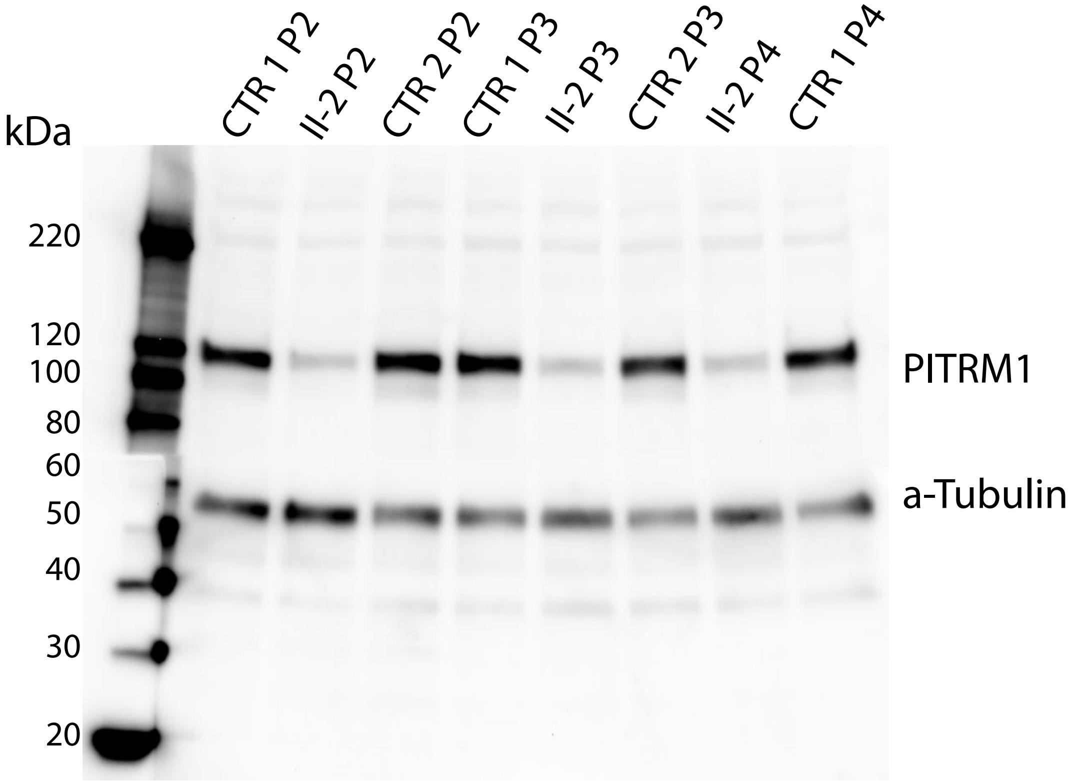

WB #2 (used in figure)

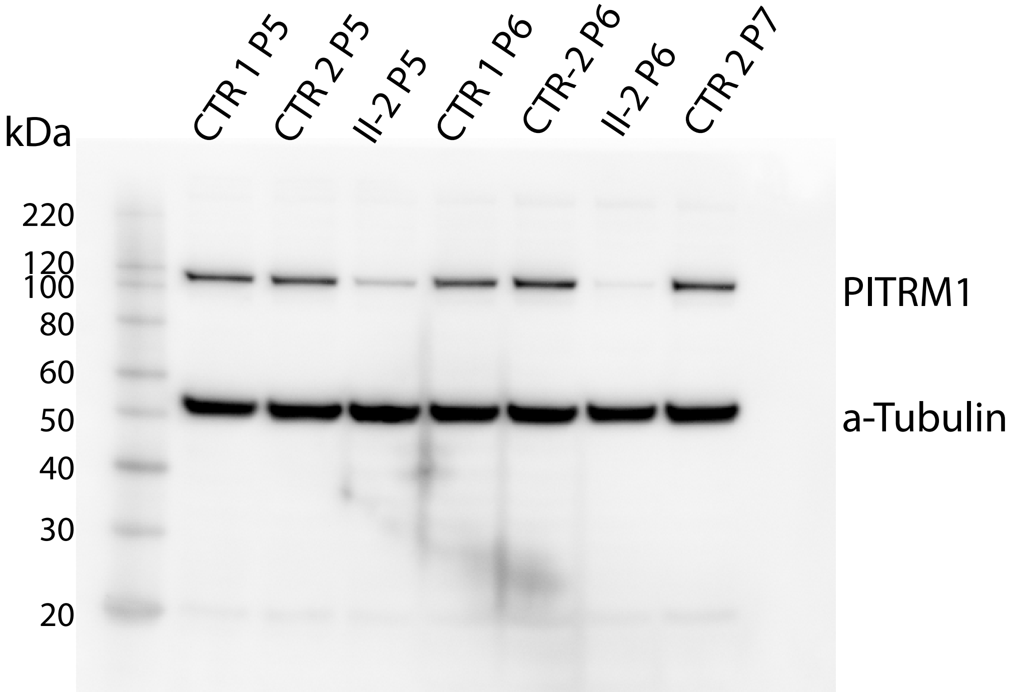

Figure 1 - G
Source Data

SDS-PAGE, Coomassie stained

Final Figure (Fig. 1G)

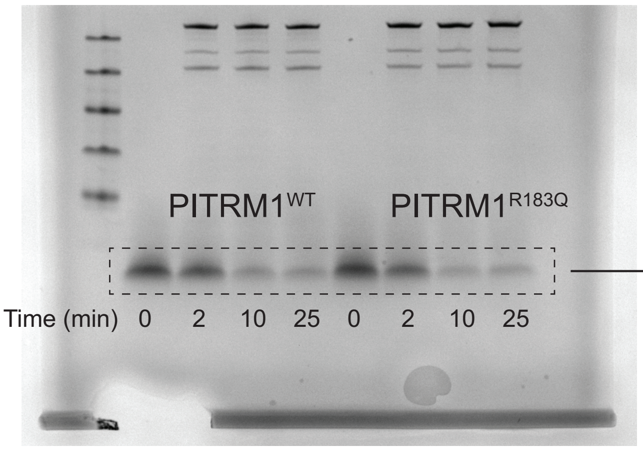

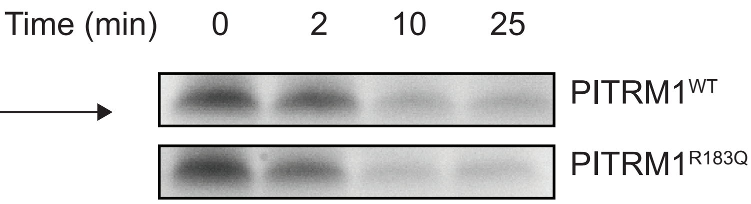

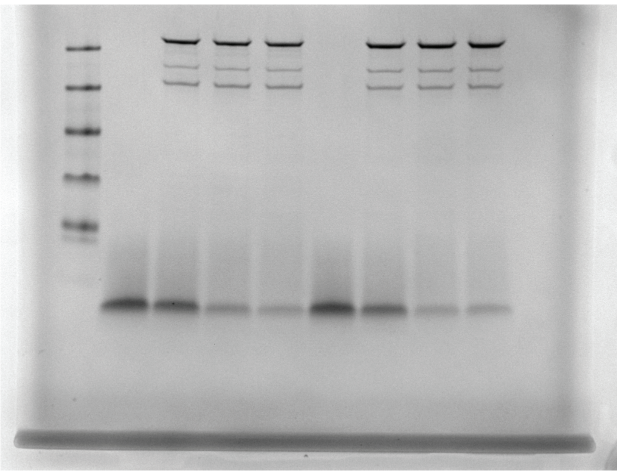

(Experiments performed in duplicate; Quantification shown in the graph in Fig. 1G)
