# Supplementary figures and images for: Defective PITRM1 mitochondrial peptidase is associated with Aβ amyloidotic neurodegeneration
Source: EMBO Mol Med. 2015 Dec 23;8(3):176–90. doi: 10.15252/emmm.201505894 (PMC4772954; doi:10.15252/emmm.201505894)

# Figure 2B

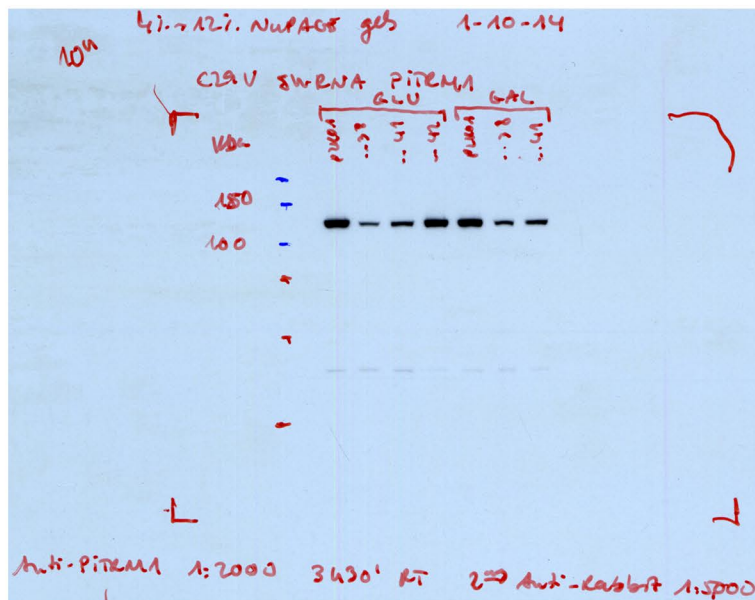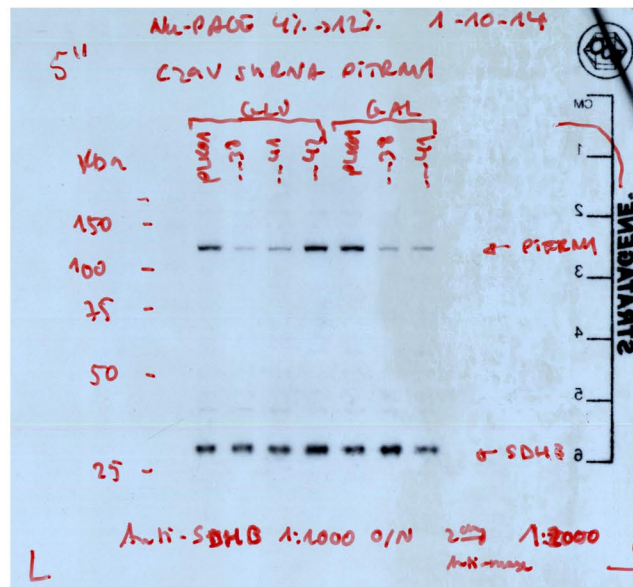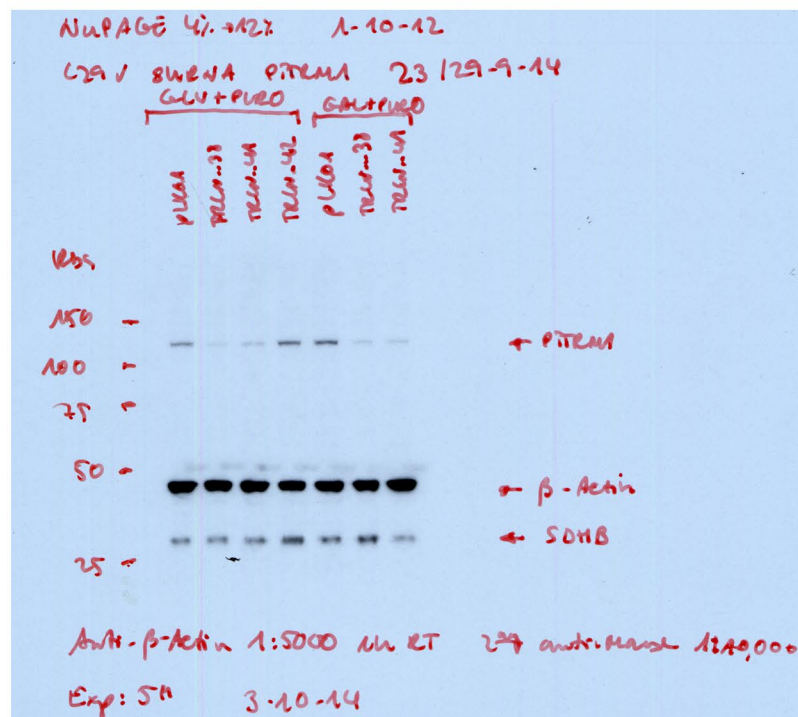

Supplement: Supplementary file 6 — Source Data for Figure 2B [file EMMM-8-176-s005.pdf]

SOURCE DATA FIGURE 3E

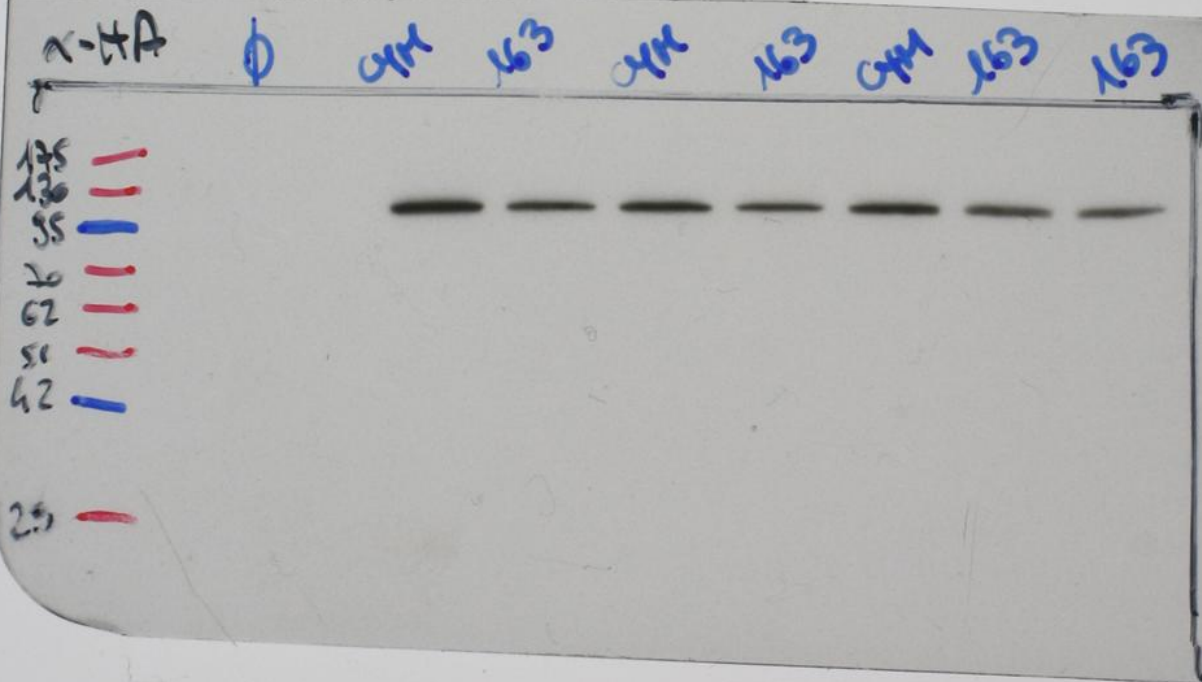



# SOURCE DATA FIGURE 3G

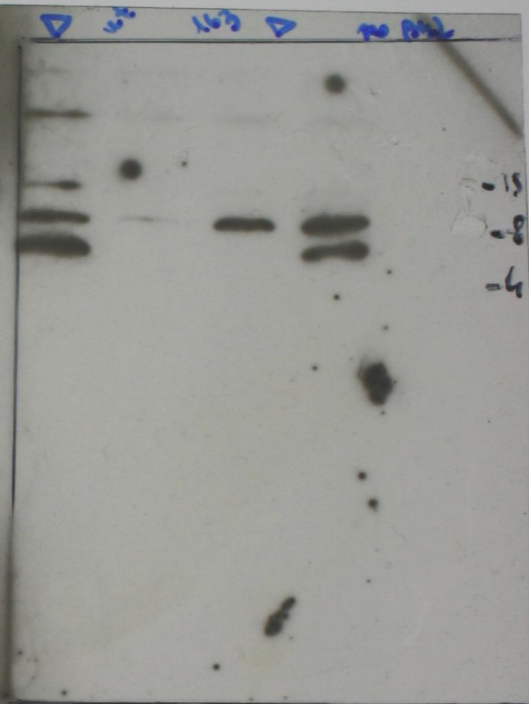

SOURCE DATA FIGURE 3G

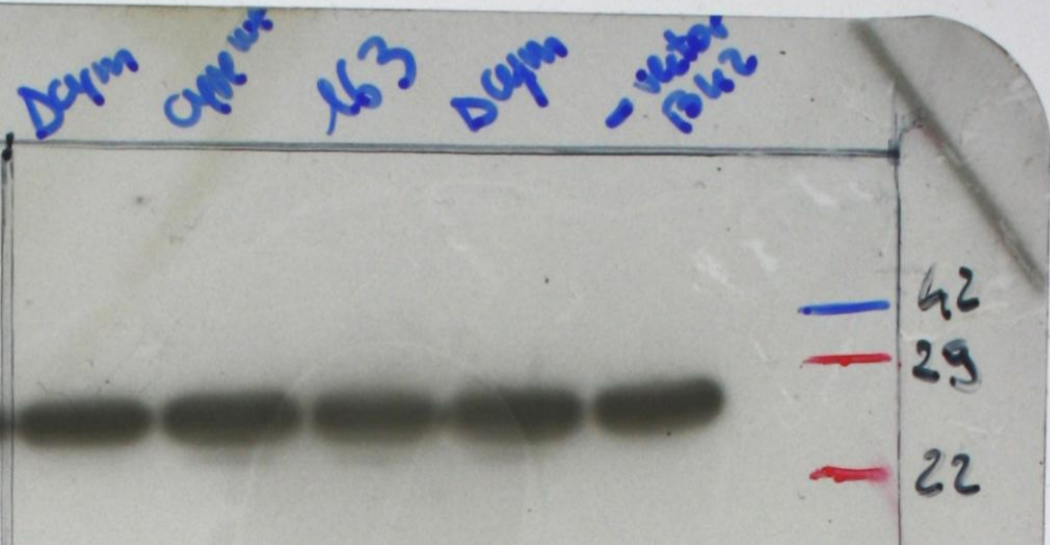

Supplement: Supplementary file 7 — Source Data for Figure 3 [file EMMM-8-176-s006.pdf]

MITO ENRICH.  
PR 10 mm

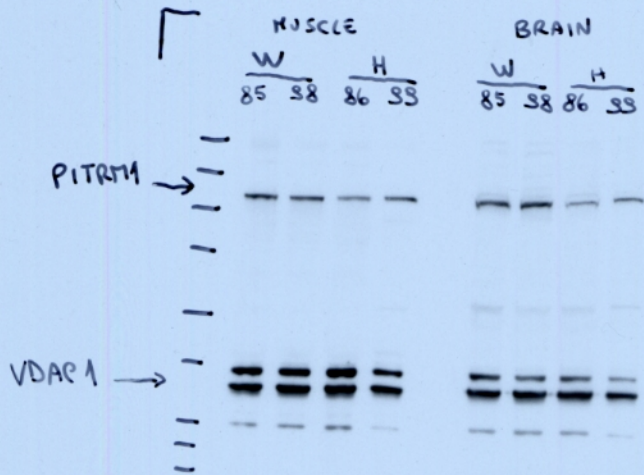

Supplement: Supplementary file 8 — Source Data for Figure 4A [file EMMM-8-176-s007.pdf]

TOT. HOMO.  
 BRAIN PITUIT (6 MONTHS)  
 APP

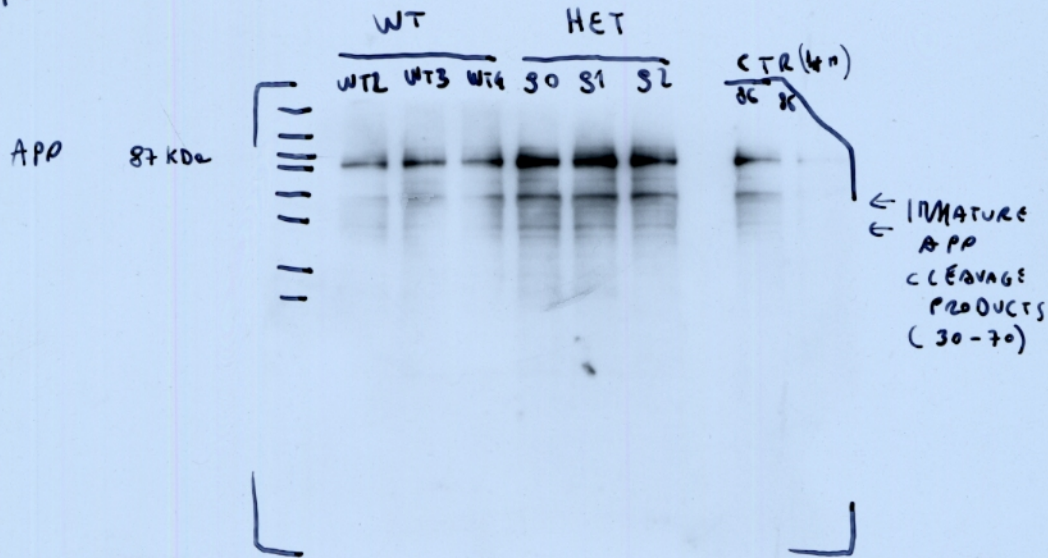

BRAIN PITRNA  
TOTAL HOMOGEN. (APP)  
APP (6 months)  
GAPDH

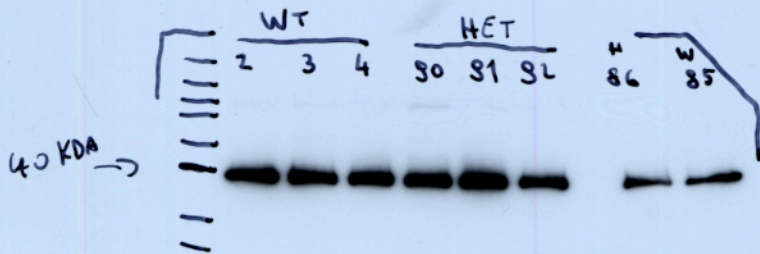

Supplement: Supplementary file 9 — Source Data for Figure 5A [file EMMM-8-176-s008.pdf]
